# Effect of Ultraviolet-C Radiation and Melatonin Stress on Biosynthesis of Antioxidant and Antidiabetic Metabolites Produced in In Vitro Callus Cultures of *Lepidium sativum* L.

**DOI:** 10.3390/ijms20071787

**Published:** 2019-04-11

**Authors:** Muhammad Asad Ullah, Duangjai Tungmunnithum, Laurine Garros, Samantha Drouet, Christophe Hano, Bilal Haider Abbasi

**Affiliations:** 1 Department of Biotechnology, Quaid-i-Azam University, Islamabad 45320, Pakistan; asad_ullah8050@yahoo.com; 2 Laboratoire de Biologie des Ligneux et des Grandes Cultures (LBLGC), INRA USC1328, Université d’Orléans, 45067 Orléans CEDEX 2, France; duangjai.tun@mahidol.ac.th (D.T.); laurine.garros@etu.univ-orleans.fr (L.G.); samantha.drouet@univ-orleans.fr (S.D.); 3 Department of Pharmaceutical Botany, Faculty of Pharmacy, Mahidol University, 447 Sri-Ayuthaya Road, Rajathevi, Bangkok 10400, Thailand; 4 Institut de Chimie Organique et Analytique (ICOA) UMR7311, Université d’Orléans-CNRS, 45067 Orléans CEDEX 2, France; 5 COSM’ACTIFS, Bioactifs et Cosmétiques, CNRS GDR3711, 45067 Orléans CEDEX 2, France; 6 EA2106 Biomolécules et Biotechnologies Végétales, Université de Tours, 37000 Tours, France

**Keywords:** polyphenols, elicitation, ultraviolet rays, secondary metabolites, antidiabetic, melatonin

## Abstract

*Lepidium sativum* L. is a rich source of polyphenols that have huge medicinal and pharmaceutical applications. In the current study, an effective abiotic elicitation strategy was designed for enhanced biosynthesis of polyphenols in callus culture of *L. sativum*. Callus was exposed to UV-C radiations for different time intervals and various concentrations of melatonin. Secondary metabolites were quantified by using high-performance liquid chromatography (HPLC). Results indicated the total secondary metabolite accumulation of nine quantified compounds was almost three fold higher (36.36 mg/g dry weight (DW)) in melatonin (20 μM) treated cultures, whereas, in response to UV-C (60 min), a 2.5 fold increase (32.33 mg/g DW) was recorded compared to control (13.94 mg/g DW). Metabolic profiling revealed the presence of three major phytochemicals, i.e., chlorogenic acid, kaemferol, and quercetin, in callus culture of *L. sativum*. Furthermore, antioxidant, antidiabetic, and enzymatic activities of callus cultures were significantly enhanced. Maximum antidiabetic activities (α-glucosidase: 57.84%; α-amylase: 62.66%) were recorded in melatonin (20 μM) treated callus cultures. Overall, melatonin proved to be an effect elicitor compared to UV-C and a positive correlation in these biological activities and phytochemical accumulation was observed. The present study provides a better comparison of both elicitors and their role in the initiation of physiological pathways for enhanced metabolites biosynthesis in vitro callus culture of *L. sativum*.

## 1. Introduction

*Lepidium sativum* L., commonly known as garden cress, is an edible herbaceous plant which belongs to the *Brassicaceae*. *L. sativum* family and is native to the Egypt and Asian regions, particularly the south west. It is being cultivated as a culinary vegetable worldwide especially in North America, India, and Europe [1,2]. *L. sativum* is also known as pepper cress and poor man’s pepper in some parts of the world. Different parts of the plant, especially the seeds, possess significant pharmaceutical properties [3]. Cress seeds have been used since prehistoric times to treat lung problems, such as cough, asthma, and bronchitis. Many studies have confirmed its potential applications as bronchodilator [4] and antirheumatic [5] agent. Cress seeds are proved to be effective against diarrhea and skin illness [6]. Studies have found the apoptotic stimulatory effects of biologically active compounds of *L. sativum* against cancer cells [7,8,9]. Seeds have been used to treat scurvy, ophthalmopathy, hemorrhoids, and leucorrhoea [10,11]. Dietary use of medicinal plants enhances the natural immunity of the body against various illnesses due to the high level of phytochemical contents. These phytochemicals, such as phenolics, flavonoids, terpenoids, and carotenoids, have therapeutic potential to protect the cells from oxidative stress which is the causative agent of some major metabolic diseases, such as cancers [12,13,14]. *L. sativum* is a rich source of these phenolic compounds including phytosterols and its derivatives that has shown anticancer, anti-inflammatory, and antioxidant potential in previous studies [15]. This medicinal species is also a rich source of essential oils [16,17]. These essential oils exhibit exceptional anticancer properties in animal models as well as cell lines as reported in the literature [18,19]. The antidiabetic potential of this plant have been described in the literature and ascribed to the presence of polyphenols [20,21].

Due to enormous medical applications, these phytochemicals are considered very important in the pharmaceutical market. Plants, in their natural environment, produce a very low quantity of secondary compounds and the downstream process is very difficult due to high variability [22]. To overcome environmental constraints and productivity issues, in vitro culture (cell, callus, shoot) provides the best alternative choice for a smooth and constant supply of plant active ingredients [23,24]. An elicitation strategy ensures high productivity for industrial-scale production of phytochemicals by initiating plant defense response via manipulation of plant physiological cascades [25,26,27]. Major categories of elicitors used in in vitro plant culture are biotic and abiotic in nature [26].

Ultraviolet light is considered an important abiotic elicitor, and has been used in phytochemical production in a variety of plant cultures in the past [28]. Exposure to UV light stress causes stimulation of defense mechanisms in plants, thus, producing commercially important secondary compounds [29]. Activation of the plant defense cascade leads to antioxidant enzymatic biosynthesis and cell modification to better adapt to the environment. Production of these compounds ensures the safety of plant cells from ROS (reactive oxygen species) generated in response to UV induced stress [30,31]. UV-C radiation (range 190–280 nm), apart from the rest of UV types, have proven to be more effective in the accumulation of phenolics, tocopherols, flavonoids, and glucosinolates [32].

Melatonin, (N-acetyl-5-methoxytryptamine), an indoleamine, is a ubiquitous and conserved molecule, found in plants as phytohormone and in vertebrates as neuro-hormone [33,34]. Primarily, melatonin in plants regulates circadian rhythms, light/dark responses and in vitro morphogenesis [35,36]. Due to the exceptional structural similarities with IAA (indole acetic acid), melatonin is reported to have a significant role in plant physiology, root development, and cell division [37,38]. The endogenous level of melatonin is largely affected by light as plants tend to accumulate a large quantity of melatonin during dark [39]. Multiple studies reported its potential role as a growth regulator against a variety of abiotic and biotic stresses [40,41]. Melatonin, when applied exogenously, plays a vital role in plant protection against cold [42], salt [43], drought [44], and copper stress [45].

The current study was designed to evaluate the potential influence of elicitors (UV-C, Melatonin) on secondary metabolic profiling of *L. sativum* callus cultures. Secondary metabolites were quantified via HPLC, and their biological potentials were studied using in vitro antioxidant and antidiabetic assays.

## 2. Results and Discussion

### 2.1. Biomass Accumulation under UV-C and Melatonin Treatment

**Figure 1:**
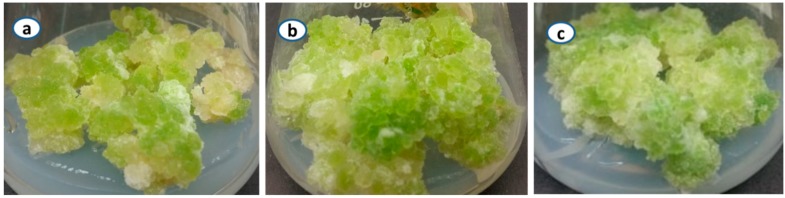
Calllus morphology of *Lepidium sativum* (**a**) Control (**b**) UV-C (60 min) (**c**) Melatonin (20 μM).

**Figure 2:**
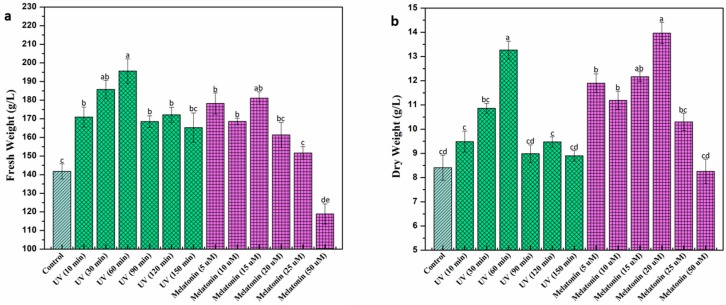
Biomass accumulation of *L. sativum* callus culture under UV-C radiations and various concentrations of melatonin. (**a**) Fresh Weight (g/L), (**b**) Dry Weight (g/L). Values are means of triplicates with the standard deviation. Columns with similar alphabets (letters a–e) are not significantly different (*p* < 0.05).

Growth pattern in callus culture of *L. sativum* under exposure to UV-C radiation and melatonin was determined by measuring its respective fresh and dry weight at given concentrations of elicitors (Figure 1). Among different exposure periods of UV-C treatment, maximum biomass accumulation (Fresh weight (FW): 195.6 mg/L, dry weight (DW): 13.26 mg/L) was recorded at 60 min UV treatment, whereas, the lowest biomass was accumulated at 150 min (FW: 165.28 mg/L DW: 8.90 mg/L), followed by control (FW: 141.79 mg/L, DW: 8.411 mg/L) (Figure 2). Longer exposure to radiations significantly reduced biomass accumulation which could possibly be due to irreversible cell damage that eventually leads to the induction of cell death [46,47]. Enhanced biomass production was previously measured in the cell culture of *Vitis vinifera* in response to 20 min exposure to UV-C radiation [48]. Different in vitro studies reported the growth stimulatory effects of UV-C radiations on medicinal plant species [46,49]. Anjum et al. [50] reported enhanced biomass production in cell cultures of *Linum usitatissimum* L. when cultures were exposed to UV-C radiation for 20 min. Furthermore, melatonin was applied exogenously to study its effects on plant growth and development. Melatonin (15 μM) showed the highest biomass production (FW; 181.05 mg/L) in callus culture compared to the rest of melatonin treatments (Figure 2a), whereas, the highest dry weight (DW; 13.966 mg/L) was recorded at melatonin (20 μM) (Figure 2b). A significant reduction in biomass was observed at higher melatonin concentrations (50 μM). Khan et al. [51] reported higher biomass production at 10 μM melatonin treatment, whereas, higher melatonin concentrations inhibited biomass accumulation. The inhibitory effect of higher melatonin concentration could possibly be due to the generation of ROS that interfere with cell growth and proliferation which leads to apoptosis [52,53]. Our study is in agreement with the previous studies [54,55] reporting inhibitory effects of higher melatonin treatments.

### 2.2. Trends in Phenolic and Flavonoid Production under UV-C and Melatonin Treatments

**Figure 3:**
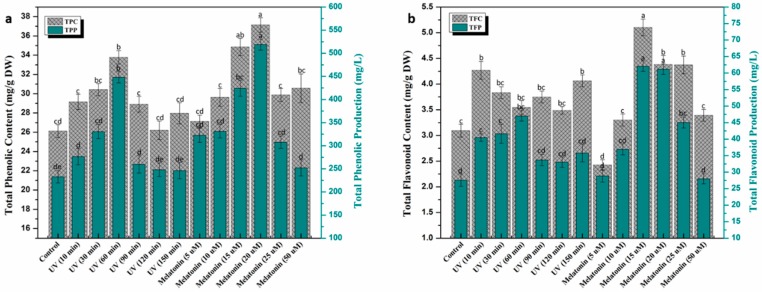
Effect of UV-C radiation and melatonin on (**a**) Total phenolic content (TPC, mg/g DW) and total phenolic production (TPP, mg/L), (**b**) Total flavonoid content (TFC, mg/g DW) and total flavonoid production (TFP, mg/L) in callus culture of *L. sativum.* Values represent means ± standard errors from triplicates. Columns with similar alphabets (letters a–e) are not significantly different (*p* < 0.05).

The current study was designed to investigate the effects of elicitors on secondary metabolites production in callus culture of *L. sativum*. Various concentrations of melatonin and UV-C doses were proved to be very effective in the biosynthesis of plant’s active ingredients. All callus cultures exposed to UV-C showed significant enhancement in phytochemical contents compared to control. Results indicated that maximum phenolics accumulation (total phenolic content (TPC): 33.77 mg/g, total phenolic production (TPP): 448.11 mg/L) was observed in UV-C (60 min), followed by UV-C (30 min) (Figure 3a) compared to rest of UV treatments, whereas, the highest total flavonoid content (TFC) (4.26 mg/g) and total flavonoid production (TFP) (47.03 mg/L) accumulation was recorded in UV-C (10 min) and UV-C (60 min), respectively (Figure 3b). The potential influence of UV-C radiation in a variety of plant species has been documented in the past [28,31,32]. UV-C radiation significantly stimulates flavonoid biosynthesis, among all phenolic compounds, due to their UV screening capacity [29,56,57]. The mechanism behind UV interaction with plant cells and secondary metabolites production is still not clear, but some studies reported the activation of CHS (chalcone synthase) and PLA (phenylalanine ammonia-lyase) enzymes in response to UV exposure, which are involved in regulation of phenolic and flavonoid biosynthesis [29,58,59]. Application of various melatonin concentrations on callus culture of *L. sativum* was also investigated for phenolic and flavonoids production. Melatonin (20 μM) greatly enhanced TPC (37.14 mg/g) and TPP (518.74 mg/L) accumulation compared to control (TPC: 26.12 mg/g, TPP: 232.81 mg/L). All melatonin treatments showed a positive effect on secondary metabolites accumulation in calli cultures (Figure 3a). Khan et al. [51] reported enhanced metabolites biosynthesis in callus culture of *Fagonia indica* in response to melatonin (10 μM). Assessment of flavonoids revealed maximum TFC (5.1 mg/g) and TFP (62.08 mg/L) accumulation at melatonin (15 μM) compared to the rest of the treatments (Figure 3b). A similar trend in flavonoids biosynthesis was reported by Khan et al. [60] in callus culture of *Ocimum basilicum* L. Melatonin has extensively been used for its growth promoting potential in in vitro derived cultures of multiple plant species [55,60,61].

### 2.3. Effect of UV-C and Melatonin on In Vitro Antioxidant Activities of L. sativum Callus

**Figure 4:**
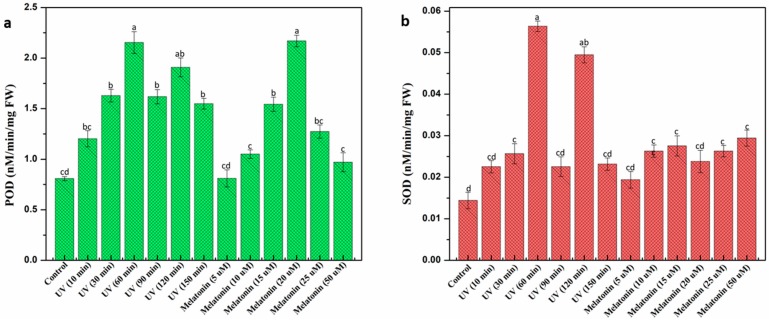
Influence of elicitors on antioxidant enzymatic activity of *L. sativum* calli extracts. (**a**) peroxidase (POD), (**b**) superoxide dismutase (SOD). Values represent means ± standard errors from triplicates. Columns with similar alphabets (letters a–d) are not significantly different (*p* < 0.05).

**Figure 5:**
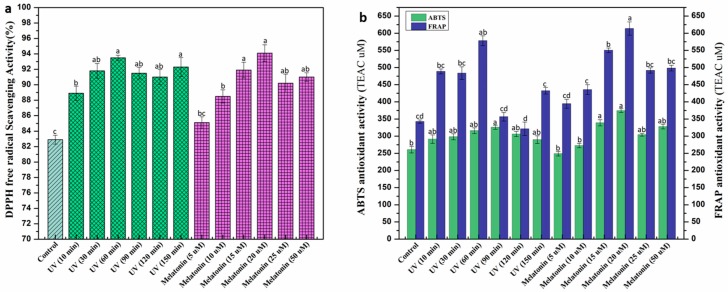
In vitro antioxidant potential of *L. sativum* callus culture (**a**) DPPH free radical scavenging activity (%), (**b**) ABTS and ferric reducing antioxidant power (FRAP) assay (TEAC: Trolox C equivalent antioxidant activity, expressed in µM). Values represent means ± standard errors from triplicates. Columns with similar alphabets (letters a–d) are not significantly different (*p* < 0.05).

Under stressed environmental conditions, plants usually produce ROS (reactive oxygen species) in large quantities that can damage cellular DNA which retards plant growth and development [62,63,64]. Plants undergo oxidative damage through the generation of free radicals, superoxide, and H_2_O_2_. Plants have a regulatory mechanism in place to combat these reactive oxygen species. Regulation of antioxidant enzymes, such as peroxidase and superoxide dismutase, are considered a key component to protect cells from oxidative toxicity in this constant battle [65,66]. The current study evaluated the potential effect of elicitors on antioxidant enzymatic activities in callus culture of *L. sativum.* Among various UV doses, optimum peroxidase (POD) activity was recorded at 60 min exposure (2.15 nM/min/mg FW) which is almost three fold higher than control (0.80 nM/min/mg FW). POD activity in response to melatonin was found higher (2.16 nM/min/mg FW) at 20 μM concentration compared to rest. A similar trend was observed in superoxide dismutase (SOD) activities (Figure 4). Overall, peroxidase activities are almost comparable in response to both UV-C and melatonin, but in the case of superoxide dismutase, UV-C radiation significantly enhanced enzymatic activity compared to melatonin. This could possibly be due to the generation of super oxides in large quantiies under stress conditions, thus, pushing plant regulatory machinery toward increased enzymatic activity [66,67,68]. Higher concentrations of melatonin were found to be inhibitory for peroxidase activity (Figure 4) but actually favorable against superoxide dismutase. These findings suggest that exogenous addition of melatonin can prove to be effective in the regulation and production of these stress enzymes [55]. To protect themselves, plants synthesize a wide range of phytochemicals including phenolics, terpenoids, and flavonoids, etc. These phytochemicals act as a natural defense against oxidative damage by mitigating ROS’s harmful effects [69,70,71]. To determine the antioxidant potential of calli extracts in response to elicitors, different in vitro based assays were employed in the current study. DPPH (2,2-diphényl-1-picrylhydrazyle) is considered as an antioxidant assay that could reveal the presence of both HAT (hydrogen atom transfer) and/or ET (electron transfer) based mechanisms for antioxidant action. Higher DPPH activity (94.1%, 93.5%) was recorded in melatonin (20 μM) and UV-C (60 min), respectively, compared to control (82.9%) (Figure 5a). Khan et al. [51] reported an elevated level of free radical activity in *Fagonia indica* treated with melatonin (10 μM), whereas, the results of Anjum et al. [50] study suggested the highest DPPH activity in *Linum usitatissimum* cell cultures exposed to UV-C (10 min). ABTS (2,2′-azino-bis-3-ethylbenzothiazoline-6-sulphonic acid, HAT based assay) and ferric reducing antioxidant power (FRAP) (ET based assay) were then used to discriminate the mechanism. Here, both mechanisms were evidenced suggesting the presence of antioxidants acting through ET based action as well as antioxidants action through a HAT based action. Results of ABTS and FRAP were expressed in TEAC (Trolox C equivalent antioxidant capacity). Melatonin (20 μM) showed maximum ABTS (374.44 μM) and FRAP (613.56 μM) activity compared to the rest of melatonin treatments (Figure 5b). Similarly, UV-C (60 min) produced optimum FRAP (578.21 μM) activity, whereas, the highest ABTS activity was observed at UV-C (90 min). FRAP results suggested that ET based action is the most prominent mechanism. The behavioral effect of the elicitor is highly influenced by the type of plant species, cell culture type, media, and growth regulators. Results of these in vitro antioxidant assays revealed a positive correlation in phenolic contents accumulation and antioxidant activity of calli extracts. Increased biosynthesis of secondary metabolites in response to elicitors might probably be the reason for the enhanced antioxidant potential of calli extracts as different studies reported similar phenomena in the past [72,73].

### 2.4. Effect of Elicitors on Antidiabetic Potential of L. sativum Callus Culture

One of the major causes of mortality and morbidity around the world is diabetes and related complications [74,75]. To study the potential influence of elicitors on antidiabetic properties of *L. sativum* extract, two enzymatic assays, i.e., solubilized α-amylase inhibition and α-glucosidase inhibition, were performed (expressed in percent inhibition activity). Callus exposure to UV-C radiations significantly enhanced its antidiabetic potential. UV-C (60 min) showed maximum α-glucosidase (48.59%) and α-amylase (55.54%) inhibition activity compared to control (25.8% and 23.41%, respectively). Our results are in accordance with Hunaefi et al. 2018 [76] reported the highest antidiabetic activity of shoot culture of *Orthosiphon aristatus* (Blume, Miq.) after 60 min exposure to UV radiation. Similarly, exogenous melatonin effect was also evaluated on the antidiabetic activity of *L. sativum*. Results revealed the highest α-glucosidase (57.84%) and α-amylase (62.66%) inhibition activity in callus extracts treated with melatonin (20 μM). Overall, melatonin stress proved to be more effective in enhancing antidiabetic activity of calli extracts compared to UV-C, which could possibly be due to a higher level of polyphenols in respective samples (Table 1). In general, callus extracts appeared to be more effective against α-amylase compared to inhibition of α-glucosidase as previously observed by Hano et al. [77] for the flax lignan secoisolariciresinol and its derivatives. The hypoglycemic effect of *L. sativum* extract has previously been studied in diabetic rats [78,79,80]. There is no study available to date, determining the effect of UV-C and melatonin on antidiabetic activity of *L. sativum* in in vitro cultures. In diabetic patients, several complications arise under hyperglycemic conditions. One of which is the formation of advanced glycation end products (AGEs) [81]. Plant phytochemicals can act as potential inhibitors for AGEs formation. Therefore, anti-AGEs formation (vesperlysine-like and pentosidine-like) activity was determined from *L. sativum* calli extracts. Optimum inhibition of vesperlysine-like (42.62%) and pentosidine-like (57.72%) AGEs formation was observed under UV-C (60 min) compared to the rest of UV treatments (Table 1). Melatonin (15 μM) appeared to be more effective against anti-pentosidine-like AGEs formation (62.36%) activity, whereas, the maximum anti-vesperlysine-like AGEs formation (52.47%) activity was observed in calli under melatonin (20 μM). In general, diabetic people show symptoms of AGEs formation in the eye lens, blood plasma, and erythrocytes [82]. Results suggest a dependent correlation between inhibition activity and polyphenol accumulation, indicating their potential use as therapeutic inhibitors against many diseases.

### 2.5. Quantification of Polyphenols Profile in L. sativum Callus Culture via HPLC

Plants tend to produce active ingredients as phytochemicals under unfavorable conditions. These phytochemicals are widely distributed throughout the plant kingdom and are essential in plant survival, defense, and development [83]. Elicitors can act as a stimulus for enhanced production of commercially important phytochemicals. Quantification of such metabolites can be done by an analytical tool, such as high-performance liquid chromatography (HPLC), with precision and accuracy. A total of nine phenolic compounds were quantified in the current study from *L. sativum* extracts exposed to UV-C and melatonin. Overall, melatonin elicited a higher level of phytochemicals compared to UV-C. Maximum secondary metabolites accumulation (36.35 mg/g) was recorded in cultures treated with melatonin (20 μM) which is almost three fold higher than control (13.94 mg/g), followed by 15 μM melatonin (32.65 mg/g) (Table 2). Similarly, UV-C (60 min) significantly enhanced phytochemical accumulation (32.33 mg/g), followed by UV-C (10 min: 27.67 mg/g) as compared to control. On the base of HPLC analysis, secondary metabolites were considered to be of two types, i.e., major and minor. Kaempferol, quercetin and chlorogenic acid are categorized as major phytochemicals accumulated in *L. sativum* callus culture. Quercetin was found higher (20.48 mg/g) in melatonin (20 μM) which is three fold higher than control (7.58 mg/g), followed by melatonin (15 μM) and UV-C (60 min) (Table 2). A similar trend was recorded in chlorogenic acid and kaempferol accumulation. Among different melatonin treatments, caffeic acid (0.915 mg/g) and ferulic acid (0.617 mg/g) were observed higher in melatonin (20 μM), whereas, vanillic acid (0.175 mg/g), sinapic acid (0.079 mg/g), and protocatechuic acid (0.057 mg/g) were found higher in melatonin (15 μM). All minor compounds showed higher accumulation at UV-C (60 min) except *p*-coumaric acid which appeared to be maximum at UV-C (10 min) (Table 2). Results of the current study revealed the significant effect of elicitors (UV and Melatonin) on the metabolic profile of *L. sativum* for enhanced phytochemical biosynthesis. A plant’s precious secondary metabolites are used against many diseases and offered a huge pharmaceutical market value. Polyphenols mediated down-regulation of oncogenes and oxidative stress damage has been reported in different studies, thus, explaining their role as anticancer, anti-inflammatory, and neuroprotective agent [84,85,86]. 

## 3. Materials and Methods

### 3.1. Chemicals

All the chemicals used in the present study were of analytical grade quality and purchased from Thermo (Illkirch, France). The deionized water was produced using a milli-Q water purification system (Merck Millipore, Molsheim, France). Before their use for analysis, all solutions were filtered through 0.45 µm nylon syringe membranes (Merck Millipore, Molsheim, France). All phytohormones and commercial standards were purchased from Sigma-Aldrich (Saint-Quentin Fallavier, France).

### 3.2. Seed Germination and Callus Induction of L. sativum

Seeds of *L. sativum* were kindly provided by PCCL (plant cell culture laboratory), Department of Biotechnology, Quaid-i-Azam University Islamabad, Pakistan. Viable seeds were separated by float test method [61] and sterilized using 30 s treatment of 0.1% mercuric chloride and 70% ethanol for 40 s, followed by three times washing with autoclaved distilled H_2_O. Seeds were placed on Murashige and Skoog (MS) medium [87] additionally fortified with 3% sucrose as a carbon source and 0.8% *w*/*v* agar for solidification. Before inoculation, medium pH was adjusted (5.6–5.8) and autoclaved at a set temperature (121 °C) for 20 min. Seeds were inoculated on a solid surfaced media and placed in a growth room under controlled conditions of photoperiod cycle (16/8 light/dark) and 25 ± 2 °C temperature.

Callus culture of *L. sativum* was established from 21 days old in vitro grown plantlets under aseptic conditions. Stem (1.0 cm) of these plantlets was excised in a laminar flow hood and transferred it to MS medium supplemented with α-naphthalene acetic acid (NAA, 1.0 mg/L) + thidiazuron (TDZ, 2.5 mg/L) as previously optimized [88]. For callogenesis, plates were transferred to the growth room at 25 ± 2 °C temperature under photoperiod cycle (16/8 light/dark). After every four weeks, callus was transferred to fresh media until a sizable callus mass was achieved for treatment of elicitors.

### 3.3. Elicitors Treatment on Callus Culture

#### 3.3.1. UV-C Treatment

Fresh calli (1 g) from sub-cultured stock was inoculated on MS media containing hormonal dose (TDZ: 2.5 mg/L and NAA: 1.0 mg/L). Callus was exposed to UV-C radiation for different exposure time (s) right after inoculation on MS media using UV-C lamp with 3 W/m^2^ radiation intensity (254 nm, Spectroline, model ZQJ-254, Hong Kong, China). Six different time durations of UV-C exposure were applied (10, 30, 60, 90, 120, 150 min) on callus culture from a fixed distance of 15 cm. Before the exposure of UV-C radiation, the UV lamp was stabilized by turning it on for 20 min approximately. Non UV treated calli was considered as control, and the whole experiment was kept in the growth room for 28 days under controlled conditions, i.e., 25 ± 2 °C and 16h/8h (light/dark) cycle. Callus was harvested after 28 days and studied for FW (g/L) and DW (g/L) accumulation.

#### 3.3.2. Melatonin Treatment

To investigate potential effect of melatonin on callus culture of *L. sativum*, fresh calli (1 g) from previously sub-cultured callus was inoculated on MS media containing hormonal dose (TDZ: 2.5 mg/L and NAA: 1.0 mg/L) additionally supplemented with different melatonin concentrations (5.0 μM, 10.0 μM, 15.0 μM, 20.0 μM, 25.0 μM, 50.0 μM). Media without melatonin was set as control. The whole experiment was placed in a growth room under controlled condition, i.e., 25 ± 2 °C and 16 h/8 h (light/dark) cycle for a 28 day time period. Callus was harvested after 28 days and studied for FW (g/L) and DW (g/L) accumulation.

### 3.4. Sample Extraction

After four weeks of elicitor exposure, callus was harvested on Whatman filter paper and gently pressed for the removal of extra water content. Fresh callus was weighed after water removal and further dried in an oven for overnight at 50 °C. Dry weight was determined, and calli were grounded into a fine powder. For antioxidant enzymatic activities (POD: peroxidase. SOD: superoxide dismutase), fresh callus sample was used as reported by Nayyar and Gupta [89]. Briefly, 0.1 g fresh calli was thoroughly homogenized with 1 mL potassium phosphate buffer (50 mM, pH 7.0) containing 1% polyvinylpyrrolidone (PVP). The reaction mixture was centrifuged for 30 min at 15,000 rpm at room temperature. The supernatant was separated into a microcentrifuge tube and stored at 4 °C for further analysis. Dry samples were subjected to extraction according to Zahir et al. [90] for estimation of secondary compounds, antioxidant, and antidiabetic activities. Briefly, 0.1 g dry powder of calli sample from each treatment was homogenized with 0.5 mL methanol, and the mixture was vortexed (5 min) and sonicated (30 min) using the USC1200TH sonicator (VWR International, Fontenay-sous-Bois, France; inner dimension: 300 mm × 240 mm × 200 mm). Callus samples were centrifuged for 15 min at 12,000 rpm, and the supernatant of each sample was separated into a microcentrifuge tube and stored at 4 °C for further analysis.

### 3.5. Estimation of Secondary Metabolites Accumulation

#### 3.5.1. Qualitative Phenolic and Flavonoids Estimation

Total phenolic contents (TPC) of elicitor-treated calli were determined by using Folin–Ciocalteu (FC) reagent as previously described by Singleton and Rossi [91]. Briefly, 90 µL of FC reagent was mixed with 90 µL of Na_2_CO_3_ and 20 µL of methanol extract of dry sample. The absorbance was taken after 5 min incubation at 725 nm wavelength using a micro-plate reader (Synergy II reader, BioTek Instruments, Colmar, France). The standard was gallic acid, and phenolic contents were expressed in terms of gallic acid equivalents (GAE)/g of dry weight. The following Formula (1) was used to calculate total phenolic production (TPP)

(1) Total phenolic production (mg/L)=DW(g/L)×TPC(mg/g)

Similarly, total flavonoid contents (TFC) were qualitatively estimated using aluminum chloride colorimetric method [52]. Briefly, 10 µL of AlCl_3_ was mixed with 10 µL of potassium acetate and 20 µL of methanol extract of dry sample, followed by 160 µL dH_2_O. The absorbance was taken after 30 min incubation time at 415 nm wavelength by a micro-plate reader (Synergy II reader, BioTek Instruments, Colmar, France). For flavonoid contents, the standard was quercetin, and flavonoids were expressed in terms of quercetin equivalents (QE)/g of dry weight. The following Formula (2) was used to calculate total flavonoid production (TFP)

(2) Total flavonoid production (mg/L)=DW(g/L)×TFC(mg/g)

#### 3.5.2. Quantitative Estimation of Polyphenols via HPLC

Metabolites quantification was done by HPLC using HPLC grade solvents and standards (Sigma Aldrich. Separation was performed on a Hypersil PEP 300 C18, 250 × 4.6 mm, 5 µm particle size equipped with a guard column Alltech, 10 × 4.1 mm (Thermo Scientific, Illkirch, France) was utilized at 35 °C and Varian a high-performance liquid chromatography system (Agilent Technology, Les Ulis, France) equipped with Varian Prostar 230 pump Meta chem Degasit, Varian Prostar 410 autosampler and Varian Prostar 335 Photodiode Array Detector (PAD) and driven by Galaxie version 1.9.3.2 software (Agilent Technology, Les Ulis, France) was used in detection of compounds at 280 and 320 nm wavelength. The mobile phase of the HPLC system was using two solvents (A: acetonitrite and B: acidified formic acid (0.1% *v*/*v*) ultrapure water). Mobile phase composition range varied during the 60 min run from 5:95 to 100:0 (solvent A:B *v*/*v*) according to a linear gradient with 0.8 mL/min flow rate. Re-equilibration time of 10 min was applied after each individual run. Quantification was done based on retention time compare to commercial reference standards (Sigma Aldrich, Saint Quentin Falavier, France). Examination of given samples was done three times, and outcomes were denoted as milligrams/gram dry weight.

### 3.6. In Vitro Antioxidant Activities

#### 3.6.1. DPPH Free Radical Scavenging Activity

The free radical scavenging potential of calli treated with elicitors was determined as previously described by Abbasi et al. [92] using DPPH reagent. Briefly, 180 µL of DPPH solution was thoroughly mixed with 20 µL of methanol extract of dry sample and the mixture was incubated in the dark for 1 h, approximately. The absorbance was taken after 1 h incubation time at 517 nm wavelength by a micro-plate reader (Synergy II reader, BioTek Instruments, Colmar, France). The negative control was set using DMSO (20 µL) with DPPH (180 µL) and the final concentrations of ascorbic acid (05, 10, 20, and 40 µg/mL). Scavenging activity was calculated by

% scavenging activity = 100 × (1 − AE/AD)

Here, AE: mixture absorbance with sample and AD: Mixture absorbance without sample.

#### 3.6.2. Ferric Reducing Antioxidant Power (FRAP) Assay

A FRAP assay was employed as previously reported by Benzie and Strain [93] to determine antioxidant capacity of calli samples. Briefly, methanol extract of dry sample (10 µL) from each treatment was mixed with FRAP solution (190 µL), composed of ferric chloride hexahydrate (20 mM), acetate buffer (300 mM) and TPTZ (10 mM) in ratio of 1:10:1 (v:v:v), respectively. Approximately, 15 min incubation period was provided to reaction mixture at 25 ± 2 °C, and the absorbance was taken at 630 nm wavelength using a micro-plate reader (Synergy II reader, BioTek Instruments, Colmar, France). For each sample, antioxidant capacity was denoted as TEAC (Trolox C equivalent antioxidant capacity).

#### 3.6.3. Antioxidant ABTS Assay

The Tagliazucchi et al. [94] method was used to determine ABTS (2,2-azinobis (3-ethylbenzthiazoline-6-sulphonic acid)) antioxidant potential of calli samples. Briefly, the ABTS solution was prepared using ABTS salt (7 mM) and potassium persulphate (2.45 mM) and placed under dark conditions for 16 h. The solution’s absorbance was taken at 734 nm and adjusted to 0.7 before adding methanol extract of dry sample. The extract was added at room temperature, and the reaction mixture was placed in the dark for 15 min after incubation, absorbance was taken at 734 nm using a micro-plate reader (Synergy II reader, BioTek Instruments, Colmar, France). For each sample, antioxidant capacity was denoted as TEAC (Trolox C equivalent antioxidant capacity).

#### 3.6.4. Anti-AGEs Formation Activity

The protocol proposed by Kaewseejan and Siriamornpun [95] for the inhibitory potential of calli samples against AGEs formation (advanced glycation end products) was used. Briefly, extracts were prepared at a concentration of 50 µg/mL in DMSO mixed with 20 mg/mL BSA (Sigma, Saint Quentin Falavier, France) and 0.5 M glucose (Sigma, Saint Quentin Falavier, France) solution; both were prepared in phosphate buffer and 1 mL of 0.1 M phosphate buffer containing 0.02% (*w*/*v*) sodium azide at pH 7.4. A VersaFluor fluorescent spectrometer (Bio-Rad, Marnes-la-Coquette, France) was used to determine AGEs formation after incubation of mixture in the dark for 5 days at room temperature with an excitation wavelength set at 330 nm and emission wavelength set at 410 nm. Anti-AGEs formation for each sample was expressed in terms of percent inhibition with respect to the relative control (same volume addition of DMSO).

### 3.7. Estimation of Antidiabetic Potential

#### 3.7.1. α-Glucosidase Inhibition

Rat intestinal acetone powder (Sigma, Saint Quentin Falavier, France) was used to purify α-glucosidase and immobilized on CNBr-activated sepharose 4B (Sigma, Saint Quentin Falavier, France) was done by a previously established protocol [77]. Briefly, 0.45 µm polyethylene filter with end-capped column was used to determine immobilized enzymatic activity by employing the chromogenic method as previously described [77]. Intestinal fluid (1 mL) was used to perform this assay containing 4-nitrophenyl-α-d-glucopyranoside (5 mM, 4NPG; Sigma, Saint Quentin Falavier, France). By adding 1 M sodium carbonate after half hour incubation time, the reaction was stopped by column filtration. Absorbance was taken at 405 nm to determine enzymatic activity against blank solution. The difference in absorbance values in the absence and presence of calli sample extracts was expressed as percentage inhibition.

#### 3.7.2. α-Amylase Inhibition

To determine soluble a-amylase enzymatic activity of calli samples, a previously described method [77] was used, and α-amylase from porcine pancreas was purchased from Sigma. In brief, phosphate buffer (pH 6.8, 0.1 M) was used to prepare enzyme at 1 u/mL concentration and 4-nitrophenyl-α-d-maltopentaoside (5 mM, 4 NPM; Sigma) was thoroughly mixed with it. The incubation period (30 min) was provided to the reaction mixture at room temperature in absence and presence of extract. The reaction was halted with sodium carbonate (1 M) solution, and absorbance was taken at 405 nm using a Synergy II reader (BioTek Instruments, Colmar, France) to determine enzymatic activity against blank solution. The difference in absorbance values in the absence and presence of calli sample extracts was expressed as percent inhibition.

### 3.8. In Vitro Antioxidant Enzymatic Activities

#### 3.8.1. Peroxidase Activity (POD)

Fresh calli sample extracts as previously mentioned in Section 2.3 were used to determine antioxidant enzymatic peroxidase activity of *L. sativum* by the Lagrimini [96] method. In brief, fresh calli sample (20 µL) form each treatment was mixed with guaiacol (20 µL, 100 mM), dH_2_O (100 µL), H_2_O_2_ (20 µL, 27.5 mM), and KH_2_PO_4_ buffer (40 µL, 50 mM). Reaction mixture without extract was used as control. Absorbance was taken using a micro-plate reader Synergy II reader (BioTek Instruments, Colmar, France) at 470 nm wavelength and the following formula was used to express enzymatic activity:

A = ELC

Here, A: sample absorbance, C: enzyme concentration (nM/min/mg FW), E: extinction coefficient (6.39 mM^−1^ cm^−1^) and L: length of wall (0.25 cm).

#### 3.8.2. Superoxide Dismutase Activity (SOD)

For estimation of antioxidant enzymatic superoxide dismutase (SOD) activity, the Giannopolitis and Ries [97] method was employed with some modifications. In brief, fresh calli sample (60 µL) form each treatment was homogenized with phosphate buffer (78 µL, 50 mM), riboflavin (2 µL, 0.02 mM), methionine (20 µL, 130 mM), NBT (20 µL, 0.75 mM) and EDTA (20 µL, 1 mM). Absorbance was taken after 7 min incubation time in fluorescent light, at 660 nm using a micro-plate reader Synergy II reader (BioTek Instruments, Colmar, France) and the following formula was used to express enzymatic activity:

A = ELC

Here, A: sample absorbance, C: enzyme concentration (nM/min/mg FW), E: extinction coefficient (6.39 mM^−1^ cm^−1^) and L: length of wall (0.25 cm).

### 3.9. Statistical Analysis

All of the experiments were carried out in an organized manner, with each treatment examined thrice (biological replicates) and repeated twice (technical replicates). Origin (OriginLab Corporation, Wellesley Hills, MA, USA) software was employed for statistical analysis, and analytical data were revealed as mean ± standard deviation with the help of Microsoft Excel (Microsoft, Redmond, WA, USA). One-way analysis of variance (ANOVA) with significant difference *p* < 0.05 was used to compare the means of different treatments.

## 4. Conclusions

The current study was designed to evaluate the influence of various doses of UV-C and melatonin on *L. sativum* callus culture for enhanced biosynthesis of active secondary compounds. Overall, melatonin showed the highest polyphenols accumulation compared to UV-C radiation. Among all UV-C treatments, radiation exposure for 60 min produced optimum results in terms of secondary metabolites accumulation, in vitro antioxidant and antidiabetic activities, whereas, melatonin (20 μM) showed higher polyphenol accumulation and biological activities as compared to other melatonin concentrations. A positive correlation in biological and antioxidant activities was observed with polyphenols accumulation. The results of the present study suggest the applicability of exogenous melatonin and UV radiations on *L. sativum* callus culture could be an effective strategy to elicit secondary metabolism of the plant.

## Figures and Tables

**Table 1:** Influence of elicitors on anti-AGEs formation and antidiabetic potential of *L. sativum* calli extracts. Columns with similar alphabets (letters a–e) are not significantly different (*p* < 0.05).

Treatment	Conconcentrations	Inhibition of Advanced Glycation End Products Formation (AGE)		Antidiabetic Activity	
		Vesperlysine-Like AGEs (% Inhibition)	Pentosidine-Like AGEs (% Inhibition)	α-Glucosidase (% Inhibition)	α-Amylase (% Inhibition)
Control	TDZ+NAA	24.28 ± 1.057 ^cd^	28.96 ± 1.444 ^d^	25.83 ± 1.057 ^d^	23.41 ± 1.443 ^e^
UV-C (min)	10	35.89 ± 0.835 ^bc^	52.15 ± 2.018 ^b^	37.86 ± 0.835 ^c^	47.34 ± 2.018 ^bc^
	30	37.45 ± 1.994 ^bc^	51.44 ± 1.702 ^b^	36.93 ± 1.999 ^c^	46.80 ± 1.726 ^bc^
	60	42.62 ± 1.520 ^b^	57.72 ± 3.101 ^ab^	48.59 ± 1.528 ^b^	55.54 ± 0.942 ^b^
	90	30.18 ± 2.018 ^c^	38.02 ± 1.973 ^c^	41.55 ± 2.018 ^bc^	35.47 ± 1.953 ^d^
	120	27.11 ± 1.720 ^cd^	33.77 ± 1.093 ^cd^	26.96 ± 1.720 ^d^	31.75 ± 1.049 ^d^
	150	36.75 ± 0.998 ^bc^	47.51 ± 1.057 ^b^	32.38 ± 0.998 ^cd^	43.50 ± 1.033 ^c^
Melatonin (μM)	5	33.45 ± 1.951 ^bc^	42.95 ± 0.845 ^bc^	27.49 ± 1.952 ^d^	39.49 ± 0.835 ^cd^
	10	37.02 ± 1.048 ^bc^	48.07 ± 1.992 ^b^	33.29 ± 1.047 ^cd^	43.85 ± 1.839 ^c^
	15	46.95 ± 2.060 ^ab^	62.36 ± 1.711 ^a^	49.55 ± 2.060 ^b^	55.98 ± 1.528 ^b^
	20	52.47 ± 2.593 ^a^	57.01 ± 1.570 ^ab^	57.84 ± 2.591 ^a^	62.66 ± 1.720 ^a^
	25	31.88 ± 1.668 ^c^	54.87 ± 0.995 ^ab^	40.73 ± 1.668 ^bc^	49.75 ± 0.936 ^bc^
	50	42.46 ± 0.946 ^b^	55.70 ± 1.947 ^ab^	41.68 ± 0.942 ^bc^	50.46 ± 1.793 ^bc^

Values are means ± SD from triplicates.

**Table 2:** Effect of UV-C and Melatonin on accumulation of phenylpropanoids in callus cultures of *L. sativum*. Columns with similar alphabets (letters a–e) are not significantly different (*p* < 0.05).

Treatment	Concentration	Polyphenolic Compounds (mg/g DW)									
		Caffeic Acid	Ferulic Acid	Vanillic Acid	*p*-Coumaric Acid	Sinapic Acid	Protocatechuic Acid	Chlorogenic Acid	Quercetin	Kaempferol	Total
Control	TDZ+NAA	0.323 ± 0.01 ^cd^	0.224 ± 0.007 ^bc^	0.076 ± 0.003 ^c^	0.129 ± 0.034 ^b^	0.044 ± 0.009 ^bc^	0.037 ± 0.0021 ^c^	2.86 ± 0.63 ^de^	7.58 ± 0.941 ^d^	2.64 ± 0.573 ^e^	13.94
UV-C (min)	10	0.682 ± 0.02 ^b^	0.462 ± 0.04 ^ab^	0.140 ± 0.005 ^ab^	0.158 ± 0.025 ^a^	0.065 ± 0.007 ^ab^	0.049 ± 0.0047 ^b^	5.24 ± 0.84 ^bc^	15.43 ± 1.843 ^b^	5.42 ± 1.053 ^bc^	27.67
	30	0.675 ± 0.009 ^b^	0.458 ± 0.027 ^ab^	0.135 ± 0.019 ^b^	0.129 ± 0.019 ^b^	0.063 ± 0.003 ^ab^	0.048 ± 0.0085 ^b^	5.17 ± 0.93 ^bc^	15.26 ± 1.909 ^b^	5.36 ± 0.593 ^bc^	27.32
	60	0.806 ± 0.06 ^ab^	0.545 ± 0.017 ^a^	0.158 ± 0.006 ^a^	0.135 ± 0.064 ^ab^	0.071 ± 0.005 ^a^	0.052 ± 0.0031 ^ab^	6.04 ± 0.48 ^ab^	18.13 ± 2.015 ^ab^	6.37 ± 1.953 ^ab^	32.33
	90	0.506 ± 0.03 ^bc^	0.345 ± 0.013 ^b^	0.103 ± 0.004 ^bc^	0.077 ± 0.004 ^c^	0.052 ± 0.001 ^b^	0.041 ± 0.0016 ^bc^	4.04 ± 0.41 ^cd^	11.56 ± 0.931 ^c^	4.05 ± 0.351 ^d^	20.78
	120	0.448 ± 0.024 ^c^	0.307 ± 0.038 ^b^	0.099 ± 0.001 ^c^	0.122 ± 0.03 ^b^	0.052 ± 0.004 ^b^	0.042 ± 0.0063 ^bc^	3.70 ± 0.52 ^cd^	10.32 ± 0.683 ^cd^	3.61 ± 0.683 ^de^	18.72
	150	0.625 ± 0.043 ^b^	0.425 ± 0.047 ^ab^	0.130 ± 0.049 ^b^	0.066 ± 0.005 ^c^	0.062 ± 0.008 ^ab^	0.047 ± 0.0074 ^b^	4.87 ± 0.48 ^bc^	14.19 ± 0.946 ^bc^	4.98 ± 0.395 ^c^	25.40
Melatonin (μM)	5	0.563 ± 0.053 ^bc^	0.384 ± 0.042 ^b^	0.123 ± 0.031 ^b^	0.155 ± 0.029 ^a^	0.060 ± 0.004 ^ab^	0.047 ± 0.0023 ^b^	4.48 ± 0.17 ^c^	12.85 ± 1.042 ^c^	4.50 ± 0.445 ^cd^	23.19
	10	0.629 ± 0.062 ^b^	0.427 ± 0.009 ^ab^	0.132 ± 0.002 ^b^	0.175 ± 0.006 ^a^	0.063 ± 0.002 ^ab^	0.048 ± 0.0019 ^b^	4.90 ± 0.52 ^bc^	14.28 ± 2.19 ^bc^	5.01 ± 1.053 ^c^	25.69
	15	0.809 ± 0.061 ^ab^	0.548 ± 0.053 ^a^	0.175 ± 0.035 ^a^	0.140 ± 0.018 ^ab^	0.079 ± 0.006 ^a^	0.057 ± 0.0067 ^a^	6.15 ± 0.201 ^ab^	18.26 ± 0.538 ^ab^	6.41 ± 1.539 ^ab^	32.65
	20	0.915 ± 0.028 ^a^	0.617 ± 0.073 ^a^	0.168 ± 0.02 ^a^	0.116 ± 0.004 ^b^	0.073 ± 0.007 ^a^	0.053 ± 0.0043 ^ab^	6.72 ± 0.42 ^a^	20.48 ± 2.638 ^a^	7.21 ± 0.213 ^a^	36.35
	25	0.719 ± 0.083 ^b^	0.487 ± 0.041 ^ab^	0.145 ± 0.018 ^ab^	0.082 ± 0.001 ^bc^	0.067 ± 0.009 ^ab^	0.050 ± 0.0064 ^ab^	5.48 ± 0.106 ^b^	16.24 ± 1.503 ^b^	5.70 ± 0.573 ^b^	28.99
	50	0.730 ± 0.042 ^b^	0.494 ± 0.098 ^ab^	0.147 ± 0.007 ^ab^	0.082 ± 0.003 ^bc^	0.067 ± 0.001 ^ab^	0.050 ± 0.0028 ^ab^	5.55 ± 0.113 ^b^	16.47 ± 1.548 ^b^	5.79 ± 0.443 ^b^	29.39
